# Microwave treatment of faecal sludge from intensively used toilets in the slums of Nairobi, Kenya

**DOI:** 10.1016/j.jenvman.2016.10.019

**Published:** 2016-12-15

**Authors:** Peter M. Mawioo, Christine M. Hooijmans, Hector A. Garcia, Damir Brdjanovic

**Affiliations:** a Department of Environmental Engineering and Water Technology, UNESCO-IHE Institute for Water Education, Westvest 7, 2611 AX Delft, The Netherlands; b Department of Biotechnology, Delft University of Technology, Julianalaan 67, 2628 BC Delft, The Netherlands

**Keywords:** Emergency sanitation, Faecal sludge, Microwave treatment, *E. coli and Ascaris* reduction, Volume reduction

## Abstract

Toilet facilities in highly dense areas such as the slum and emergency settlements fill up rapidly; thus, requiring frequent emptying. Consequently, big quantities of fresh faecal sludge (FS) containing large amounts of pathogens are generated. Fast and efficient FS treatment technologies are therefore required for safe treatment and disposal of the FS in such conditions. This study explores the applicability of a microwave (MW) technology for the treatment of fresh FS obtained from urine-diverting dry toilets placed in slum settlements in Nairobi, Kenya. Two sample fractions containing 100 g and 200 g of FS were exposed to MW irradiation at three input MW power levels of 465, 1085 and 1550 W at different exposure times ranging from 0.5 to 14 min. The variation in the FS temperature, pathogen reduction via the destruction of *E. coli* and *Ascaris lumbricoides* eggs, and vol/wt reduction were measured during the MW treatment. It was demonstrated that the MW technology can rapidly and efficiently achieve complete reduction of *E. coli* and *Ascaris lumbricoides* eggs, and over 70% vol/wt reduction in the fresh FS. Furthermore, the successful evaluation of the MW technology under real field conditions demonstrated that MW irradiation can be applied for rapid treatment of fresh FS in situations such as urban slum and emergency conditions.

## Highlights

• There is lack of appropriate options to treat sludge from intensively used toilets.
• Microwave treatment is fast and efficient in sludge volume and pathogen reduction.
• Microwave technology can be applied to treat fecal sludge in slums and emergencies.

## Graphical abstract

**Figure:**
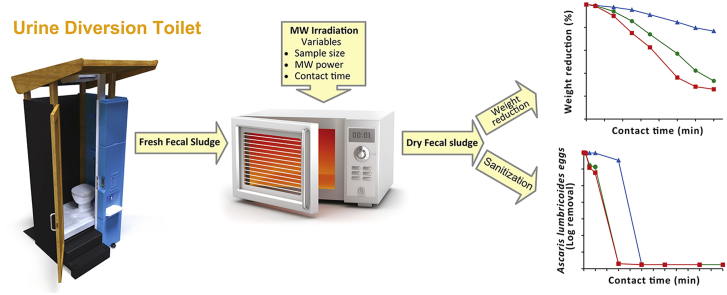

## 1 Introduction

Sanitation facilities, especially the toilets provided in densely populated areas, such as urban slums and emergency settlements, fill up fast due to intensive use and they require frequent emptying. For instance, Sanergy, a social enterprise, empties fresh faecal sludge (FS) from over 700 toilet units that serve over 30,000 users daily in the informal settlements of Nairobi, Kenya (Sanergy, personal communication). Also, approximately 50–200 users per toilet per day are commonly observed in disaster situations (The Sphere Project, 2011); especially, at the onset of emergencies (UNHCR, personal communication). Consequently, large quantities of fresh FS are generated which require safe treatment and disposal. Various issues are identified that generally present a challenge to the FS management, especially in densely populated conditions. FS contains high amounts of pathogens such as bacteria, helminths, viruses, protozoa, and others (Fidjeland et al., 2013, Jimenez et al., 2006, Richard, 2001), which can pose a great risk to the public heath if it is inappropriately managed. In addition, the large amount of emptied fresh FS may need to be transferred to disposal sites far away from the points of generation, as in slum and emergency settlements where land space constraints and lack of adequate disposal possibilities are common. Massive expenditure may thus be incurred in emptying and transporting large amounts of FS, which would make the operation and maintenance of the sanitation system overly expensive. An example is the Haiti emergency camps six months after the 2010 earthquake, where a relief agency (Action Contre la Faim (ACF)) still incurred a monthly expenditure of approximately USD 500,000 to empty the toilets and dispose of FS (Bastable and Lamb, 2012). FS also contains high amounts of organic matter whose uncontrolled degradation in the environment can result in the generation of offensive odour, which may cause respiratory-related complications and attract disease vectors. These concerns form a major challenge to FS management in densely populated areas; hence requiring solutions that are more adapted to those conditions. The common sanitation solutions provided in urban slum and emergency settlements are mainly containment options. These comprise a range of onsite toilet facilities including chemical toilets, packet toilets (e.g. peepoo and wagbag), bucket latrines or elevated toilets, trench latrines, pit latrines, and others (Harvey, 2007, Katukiza et al., 2012). In recent years, there have also been remarkable efforts to expand the onsite toilet options in which a number of prototypes have been developed. However, parallel efforts to develop technology options to treat FS generated from those toilets still have to be demonstrated in practice.

Various treatment alternatives are available for FS such as composting, co-composting with organic solid waste, conventional drying (e.g. in sludge drying beds), anaerobic co-digestion with organic solid waste, and co-treatment in wastewater treatment plants (Ingallinella et al., 2002, Katukiza et al., 2012, Ronteltap et al., 2014). However, they are mostly suited for regular sanitation contexts and have limitations such as slow treatment processes, large land space requirements, among others (Mawioo et al., 2016), which hinder their application to situations with unusually high rate of FS generation. Consequently, there is need to develop FS treatment technologies that are more appropriate for conditions such as those prevailing in slums and emergencies. Key among the desired characteristics for an appropriate FS treatment technology in these situations is that it should be fast, efficient, and compact for easy and rapid deployment. Particularly, the technology should as much as possible address the various issues of concern mentioned above. In those areas with a high generation rate of fresh FS (e.g. slums and emergencies), the reduction of pathogenic organisms should definitely be prioritized over sludge volume and organic matter reduction so as to minimize the risk of excreta-related disease outbreaks. The amount of the pathogenic organisms should be reduced to the recommended safe levels (e.g. *E. coli* to ≤1000 CFU/g TS and *Ascaris* eggs to <1 *Ascaris* egg/g TS (WHO, 2006)). Next to pathogen reduction, it is often desirable to reduce the FS volume (to minimize handling costs) and organic matter content (to avoid odour and disease vector nuisance).

A microwave (MW) based technology can be a viable option for the treatment of fresh FS from intensively used toilet facilities as it has been shown being regarding the efficient pathogen inactivation and volume reduction (Mawioo et al., 2016). MW irradiation uses the MW energy (E_MW_) with wavelengths between 1 mm and 1 m and frequencies between 300 MHz and 300 GHz in the electromagnetic spectrum (Haque, 1999, Remya and Lin, 2011, Tang et al., 2010). The MW technology has been used in various applications, most of which involve the use of heat generation. In such applications, the heat is generated by the molecular motion in the target material resulting from the migration of ionic species and/or rotation of the dipolar species when they interact with the microwaves (Haque, 1999, Mawioo et al., 2016, Thostenson and Chou, 1999). Various benefits are associated with heating by MW (Haque, 1999, Mawioo et al., 2016). The heating of a material by microwaves depends on its dielectric properties (i.e. the dielectric loss factor and the dielectric constant) and materials with high dielectric loss factor are favorable for the MW heating. Various types of sludge, such as sewage sludge and blackwater sludge (i.e. sludge extracted from a blackwater stream generated in low flush toilets, TS = 12%) contain dipolar molecules (e.g. water and organic complexes) with high loss dielectric properties and have demonstrated a good response to the MW treatment (Mawioo et al., 2016, Yu et al., 2010). For instance, nearly complete bacterial removal was reported when sewage sludge (Hong et al., 2004, Hong et al., 2006) and blackwater sludge (Mawioo et al., 2016) were heated by MW to temperatures above 65 °C. Furthermore, over 70% sludge volume reduction was achieved by treating blackwater sludge (Mawioo et al., 2016) and anaerobic sewage sludge (Menéndez et al., 2002) with MW. The MW effect on the pathogen destruction is linked to both the non-thermal (electromagnetic radiation) and thermal (temperature) effects of electromagnetic energy (Banik et al., 2003, Hong et al., 2004, Mawioo et al., 2016). By electromagnetic radiation, molecules of the irradiated material orient themselves in the direction of the electric field, which may break the hydrogen bonds leading to the denaturation and death of microbial cells (Banik et al., 2003, Tyagi and Lo, 2013). Conversely, the destruction by thermal effect is caused by the rapturing of microbial cells when water is rapidly heated to the boiling point by rotating dipole molecules under an oscillating electromagnetic field (Hong et al., 2004, Tang et al., 2010, Tyagi and Lo, 2013). On the other hand, volume reduction is strongly linked to the temperature increase, which causes evaporation of the water contained in the sludge (Mawioo et al., 2016). Stabilization of organic matter in sludge was not achieved by MW heating, arguably due to the relatively low maximum temperature (i.e. 127 °C) attained in the treatment process (Mawioo et al., 2016). However, organic stabilization was achieved by Menéndez et al. (2002) when they mixed sludge with a MW receptor material to attain high temperatures (over 900 °C).

As discussed above, waste management by MW technology has been demonstrated through treating the various kinds of sludge. The technology possesses a rapid heating and treatment capability, which can be explored further for possible applications in treating fresh FS in slum and emergency settlements. Despite the reported successes in the various kinds of waste treatment, the information on the evaluation of the MW technology in FS treatment for a potential field application is still limited. So far, only a recent study evaluating the MW treatment of blackwater sludge has been reported; in which *E. coli*, sludge volume, and organic matter reduction was assessed (Mawioo et al., 2016). It is therefore highly needed to evaluate the potential application of this technology for specific field applications using fresh FS obtained from toilet facilities under real field conditions. In addition, the previous studies demonstrated pathogen reduction on *E. coli* and faecal coliforms; therefore, including other pathogenic organisms such as helminth eggs is also important, as they are shown to be more resistant to treatment (Feachem et al., 1983; Koné et al., 2007, WHO, 2006). If successful, the information derived from such study can help to further validate the MW application for treatment of FS under those conditions and set the basis for scaling up the technology. Also, the study can expand knowledge about the response of different types of sludge to MW treatment.

In this study the potential of a MW based technology for slum or emergency sanitation applications was evaluated by treating fresh FS obtained from toilets in the slums of Mukuru and Mathare in Nairobi, Kenya. Besides being a representation of FS in an urban slum environment, the fresh FS sample obtained in these conditions is also relatively similar to that which is generated in emergency camps. Three aspects of the proposed MW treatment technology were assessed in this research including the reduction of pathogens, sludge volume, and organic matter. Both *E. coli* and helminth (*Ascaris lumbricoides*) eggs were used as indicators for pathogen reduction, while the sludge weight was used to estimate the volume reduction. The organic stabilization of the FS was estimated using the volatile and total solids ratio (VS/TS) as indicator.

## 2 Materials and methods

### 2.1 Research design

This study was performed using fresh FS samples obtained from Fresh Life^®^ toilets, which are installed and maintained by Sanergy in collaboration with entrepreneurs in the slums of Nairobi, Kenya. Fresh Life^®^ is the brand name of Sanergy toilets that uses the principle of urine-diverting dry toilet (i.e. faeces and urine streams are diverted and collected in separate containers). The toilets are emptied on a daily basis by removing and replacing the filled containers with clean empty ones. Over seven metric tons of FS is then transported to a central treatment facility (approximately 30 km from Nairobi), where it is converted into fertilizer via composting. The ultimate goal of this study was to test the MW technology for treatment of fresh FS generated from intensively used toilet facilities in areas such as slums and emergencies. Therefore, the Fresh Life^®^ toilets were chosen for the FS source because besides their location in a slum settlement, they are also comparable to the emergency toilet facilities in many aspects. For instance, the dry (i.e. non flush) toilet systems, similar to the Fresh Life^®^ toilets, are the most common FS management technologies applied in emergency camps (Harvey, 2007). Furthermore, due to the high population densities in the slum settlements, the intensive toilet usage and corresponding rapid fill up is similar to that encountered in emergency camps. These similarities allowed the study to be also relevant to the emergency conditions. All of the necessary research infrastructures (e.g. specialized FS laboratory and expert support) was made available to the research team through funding from the Bill & Melinda Gates Foundation.

During the study, two amounts of FS (i.e. 100 and 200 g) were treated by exposing it to MW irradiation in a domestic MW oven for various durations and input MW power levels. The changes in the various parameters including temperature, *E. coli*, helminths (*Ascaris lumbricoides)* eggs, weight, and VS/TS ratio were measured in the treated samples. The experiments for both the 100 g and 200 g samples were conducted in triplicates and repeated to obtain three trials. The effectiveness of the MW treatment was then determined by the changes in the measured parameters between the raw and the treated FS samples (Mawioo et al., 2016).

### 2.2 Microwave apparatus

A domestic microwave oven, Samsung, MX245 (Samsung Electronics Benelux B.V., the Netherlands) was used in this study. The unit operates at a frequency of 2450 MHz with a power output ranging from 0 to 1550 W with 10% incremental steps. The microwave oven was placed in a makeshift structure that was located at the Sanergy's central waste treatment facility in Nairobi, Kenya where the entire research activities were carried out.

### 2.3 FS samples

Containers with FS from three toilets were identified from which approximately equal, but large, portions of the fresh FS samples were obtained and transferred into a plastic bucket. The samples were then mixed thoroughly on site to attain a homogenous sample from which three smaller samples were drawn and placed into plastic sampling containers. The samples were then transported to the Sanergy's central waste treatment facility where the MW treatment experiments were conducted within 24 h. The characteristics of the fresh FS are presented in Table 1.

### 2.4 Experimental procedures

#### 2.4.1 Sample preparation

The two sizes of test samples were prepared in triplicates: the 100 g FS samples were placed in one liter glass beakers (height of the FS was approximately one centimeter, surface area approximately 78.5 cm^2^), while the 200 g samples were placed in two liter glass beakers (height of the FS was approximately one centimeter, surface area approximately 156 cm^2^). As shown in Table 1 above, the *E. coli* naturally occurring in the FS (i.e. 4.0 × 10^8^ CFU/g TS) was sufficient for evaluating the performance of the MW technology on *E. coli* inactivation; thus spiking *E. coli* was not necessary. However, an analysis of the FS samples did not reveal existence of *Ascaris lumbricoides* (helminths indicator) eggs. Therefore, the evaluated FS samples were spiked with *Ascaris lumbricoides* eggs before the MW treatment. The samples of 100 g and 200 g were spiked by adding and mixing approximately 1 × 10^4^ and 2 × 10^4^ *Ascaris lumbricoides* eggs, respectively.

#### 2.4.2 Microwave treatment

The FS samples were treated in the MW apparatus (Section 2.2). The sample contained in the glass beaker was placed in the MW cavity and then irradiated at 465, 1,085, and 1550 W for varied time durations (i.e. between 0.5 and 14 min). After the MW treatment, the sample was removed from the MW cavity and its temperature was immediately measured before covering with sanitized aluminum foil. The samples that underwent microwave treatment were cooled down to room temperature and analyzed for their characteristics as described in the following sections.

### 2.5 Analytical procedures

FS samples with and without MW treatment were measured for various physical-chemical parameters including temperature, weight, TS, and VS and the microbial parameters including the *E. coli* and *Ascaris lumbricoides* eggs.

#### 2.5.1 COD measurement

A known amount of FS sample (prior to MW treatment) was diluted in demineralized water, after which the COD concentration was measured according to the closed reflux method (SM 5220 C) (APHA, 1995) and expressed in mg COD per g TS (mg COD/g TS) (Table 1).

#### 2.5.2 Temperature

The initial sample temperature was measured just before MW treatment using an infrared thermometer (Fluke 62 MAX, Fluke Corporation, U.S.A). Following each treatment, the sample was taken out from the MW cavity and the temperature was immediately measured. Due to the solid nature of the sample, the temperature was only measured on the surface.

#### 2.5.3 Weight measurement

The initial weight was measured using a bench-top weighing balance (Sartorius H160, Sartorius AG, Germany) as the samples were transferred into the heating beakers. The final weight after MW treatment was measured once the samples were cooled to room temperature. The volume reduction was then determined from the initial and the final sample weight difference. Based on the maximum temperature attained during MW treatment (i.e. ≤ 134 °C), the weight reduction could mainly be attributed to the water evaporating from the heated sludge. Thus, considering the density of water, the weight reduction was deemed to be equivalent to the sludge volume reduction.

#### 2.5.4 TS and VS measurement

TS content was determined by drying the samples in an oven at 105 °C for at 24 h (for TS), after which they were cooled and weighed. The VS was determined in the same samples by burning in a muffle furnace at 550 °C for two hours. The TS and VS results were then used to evaluate the organic stability of sludge.

#### 2.5.5 *E. coli* measurement

The detection of *E. coli* was done using the surface plate technique with chromocult coliform agar (Chromocult; Merck, Darmstadt, Germany) (Byamukama et al., 2000). A step by step procedure similar to what is previously reported in Mawioo et al. (2016) was applied. Dark blue to violet colonies were classified as *E. coli* (Byamukama et al., 2000, Sangadkit et al., 2012). The average number of colonies were used to calculate the viable-cell concentrations in the samples, expressed in CFU/g TS of the test sample.

#### 2.5.6 Recovery of *Ascaris* eggs and incubation

The *Ascaris eggs* recovery was done according to the protocol developed by Moodley et al. (2008) and modified in Pebsworth et al. (2012). Portions (20 g) of the respective MW treated samples were placed on a clean plastic beaker to which 80 mL of ammonium bicarbonate (119 g of ammonium bicarbonate in one liter of de-ionized water) was added and then mixed on a magnetic stirrer for 20 min. The mixture was poured through a 100-μm sieve fitted on top of a 25 μm sieve (200 mm dia.) and thoroughly washed with tap water. The material retained on the 100 μm sieve was discarded while that retained on the 25-μm sieve was thoroughly washed and then rinsed into a clean plastic beaker. The solution was then transferred into 15 mL Falcon tubes and centrifuged at 3000 rpm for five minutes using a bench-top centrifuge (EBA 20, Andreas Hettich GmbH &CO. KG, Germany). The supernatant was discarded while the solid pellet containing the eggs was re-suspended by adding ZnSO_4_ (specific gravity 1.3) when vortexing until the tubes were filled to the 14 mL mark. The solution was centrifuged at 2000 rpm for five minutes and the resultant supernatant floatation fluid poured over a smaller 25 μm sieve (100 mm dia.). The material retained on the sieve was washed well with tap water and then rinsed into a clean plastic beaker. The solution was transferred back into the 15 mL plastic test tubes (Falcon) and centrifuged one last time at 3000 rpm for five minutes. The supernatant was discarded and the egg pellets (the concentrate of the centrifugation) were transferred into a 50 mL Falcon tube containing 10 mL of de-ionized water. The Falcon tube was covered with a plastic film (Parafilm) that was pricked (to allow air into the sample) and acted as a humid chamber for incubation at 28 °C for 28 days.

#### 2.5.7 Viability test and eggs count

After the 28 days incubation, the eggs were transferred to the 15 mL Falcon tubes and centrifuged for five minutes at 3000 rpm. The supernatant was removed and the remaining pellet containing eggs was well mixed using a pipette. The suspension of the eggs (1 mL) was placed on a microscopic slide and covered with a cover slip. The slide was observed under the microscope (AmScope, California, USA) at a magnification of 10 and 40. The eggs were counted as living if they contained a fully developed larva and dead without a larva but with an internal structure.

### 2.6 Data analysis

The experimental data was processed using Microsoft Excel software. Firstly, the data for each trial were separately processed by computing the average values for each set of the triplicate treatments. Then the average values obtained in each of the three trials were further combined by computing their mean values. Furthermore, the respective standard deviations and standard errors for the combined trials mean values were calculated. The mean values for the three trials were then presented in either tables or figures with their respective standard error values or error bars shown. Linear regression was performed; particularly to estimate the specific energy demand rates (i.e. watt-hour, Wh per gram) on each of the three drying phases.

## 3 Results

### 3.1 Temperature evolution

**Fig. 1:**
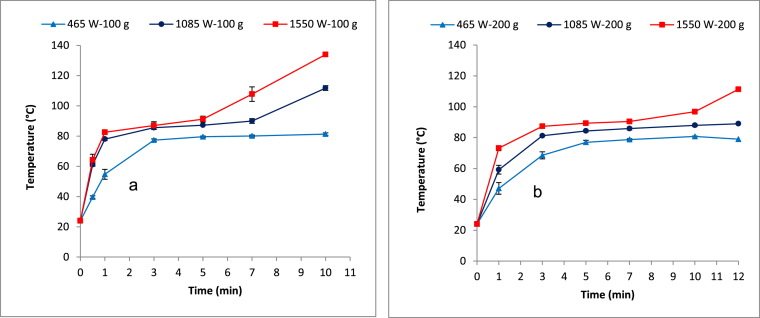
Effect of exposure to microwaves on temperature evolution in a) 100 g sample and b) 200 g sample.

The temperature profiles for the 100 g and 200 g sample during the MW treatment are shown in Fig. 1a and b, respectively. Three distinctive temperature evolution phases were observed during the respective input MW power levels and contact times evaluated. Furthermore, different temperature propagation rates were observed among the three phases. For instance, there was a rapid rise in the FS temperature during the initial phase (Fig. 1a and b) while a fairly constant and minimal rise was observed in the second phase. A rapid rise in temperature was again observed during the third phase. It is also evident from Fig. 1a and b that the temperature increment was more rapid in the 100 g than the 200 g sample in the initial phase. For instance, while after a one minute contact time the 100 g sample attained a temperature of 55, 78, and 83 °C at the respective input MW power levels of 465, 1,085, and 1550 W; the corresponding temperatures attained in the 200 g sample were lower, namely 47, 59, and 73 °C, respectively.

### 3.2 Pathogen reduction

#### 3.2.1 *E. coli* reduction

**Fig. 2:**
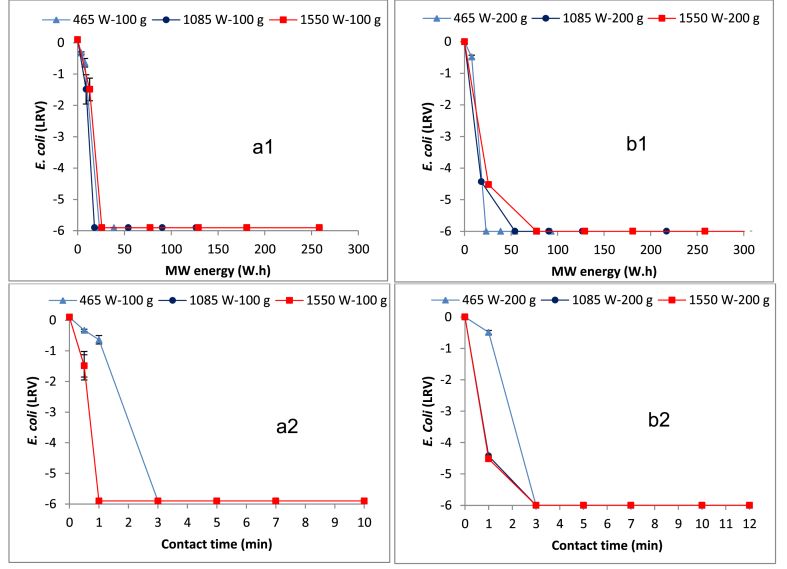
Effect of MW energy on the *E. coli* reduction in a1) 100 g FS sample and b1) 200 g FS sample, and *E. coli* reduction as a function of contact time in a2) 100 g FS sample and b2) 200 g FS sample. The zero *E. coli* log removal corresponds to an initial concentration of 4.0 × 10^8^ CFU/g TS).

Fig. 2a1, a2, and b1, b2 show the results of the *E. coli* reduction in the 100 and 200 g samples obtained at various input MW power levels and contact times, respectively. Furthermore, the influence of temperature on the *E. coli* reduction over contact time was observed. The results show increased *E. coli* reduction when the input MW power level and/or the contact time was increased. Also, as expected, there was an observed reduction of *E. coli* with the rise in the FS temperature. For instance, a reduction of *E. coli* was achieved when the 100 g sample was heated at 465 W for one minute (i.e. E_MW_ = 8 Wh, T = 55 °C) resulting to approximately 0.74 log removal value (LRV), i.e. 80% removal. However, an even higher reduction below the detection limit (i.e. <1000 CFU/g TS or approximately 5.60 LRV) was achieved when the input MW power level was raised to 1085 W (E_MW_ = 18 Wh, temp = 78 °C) and 1550 W (26 Wh, temp = 83 °C) at the one-minute contact time. A reduction below the detection limit was achieved with the 465 W when the contact time was increased to three minutes (i.e. E_MW_ = 23 Wh, T = 77 °C). A similar trend to that observed in the 100 g sample was also demonstrated in the 200 g sample (Fig. 2b1 and b2) in which the increment in the input MW power led to increased *E. coli* reduction. However, lower inactivation efficiencies were observed in the 200 g sample compared to the 100 g sample. For instance, while reduction below the detection limit was achieved with the 1085 W and 1550 W at the one-minute contact time in the 100 g sample, only approximately 4.4 LRV was achieved in the 200 g sample. Nevertheless, *E. coli* reduction below the detection limit was achieved in each evaluated input MW power at the three minutes contact time. The corresponding energy and temperature levels attained at the three minutes contact time were 23 Wh and 69 °C, 54 Wh and 81 °C, and 78 Wh and 87 °C for the 465 W, 1085 W and 1550 W, respectively.

#### 3.2.2 *Ascaris* egg reduction

**Fig. 3:**
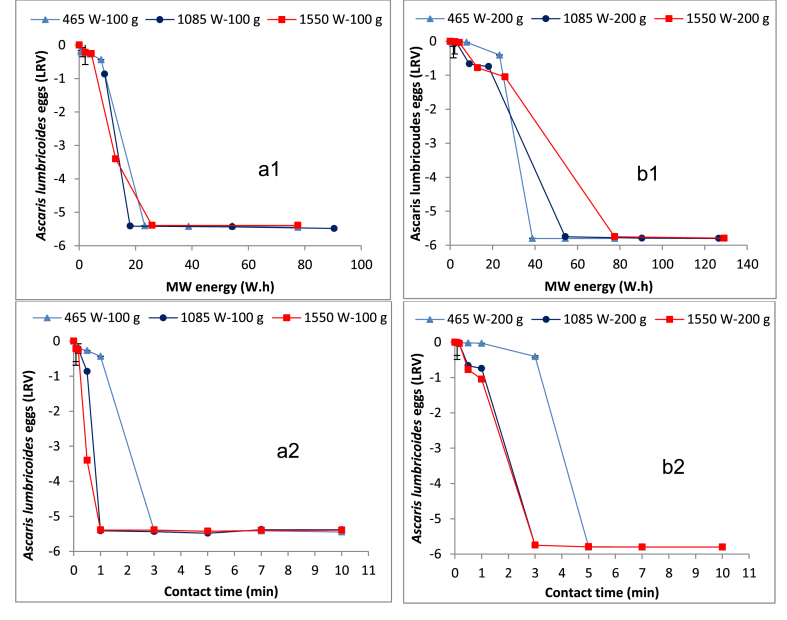
Effect of MW energy on the *Ascaris lumbricoides* reduction in a1) 100 g FS sample and b1) 200 g FS sample, and *Ascaris lumbricoides* reduction as a function of contact time in a2) 100 g FS sample and b2) 200 g FS sample. The zero *Ascaris lumbricoides* eggs log removal corresponds to an initial concentration of 2.69 × 10^2^*Ascaris* eggs/g TS.

Fig. 3a1, a2, and b1, b2 presents the profiles for *Ascaris lumbricoides* egg reduction as a function of the MW energy and the exposure time for the 100 g and 200 g FS samples, respectively. The influence of temperature on the *Ascaris* egg reduction over contact time was also observed. As shown in the figure, the MW treatment was successful in achieving over 3 LRV reduction of the *Ascaris* eggs within one minute when the 100 g sample was exposed to MW irradiation at 1085 W (E_MW_ = 18 Wh, T = 78 °C) and 1550 W (E_MW_ = 26 Wh, T = 83 °C). However, to achieve a similar reduction at 465 W, a longer contact time, at least three minutes (E_MW_ = 23 Wh, T = 77 °C) was required. On the other hand, for the 200 g sample, at least a three minute contact time was required for the 1085 W (i.e. E_MW_ = 54 Wh, T = 81 °C) and 1550 W (i.e. E_MW_ = 78 Wh, T = 87 °C) to achieve over 3 LRV reduction. However, a longer contact time (at least five minutes, i.e. E_MW_ = 39 Wh, T = 77 °C) was required to achieve similar results when 465 W was used.

### 3.3 Volume reduction and energy requirements

**Fig. 4:**
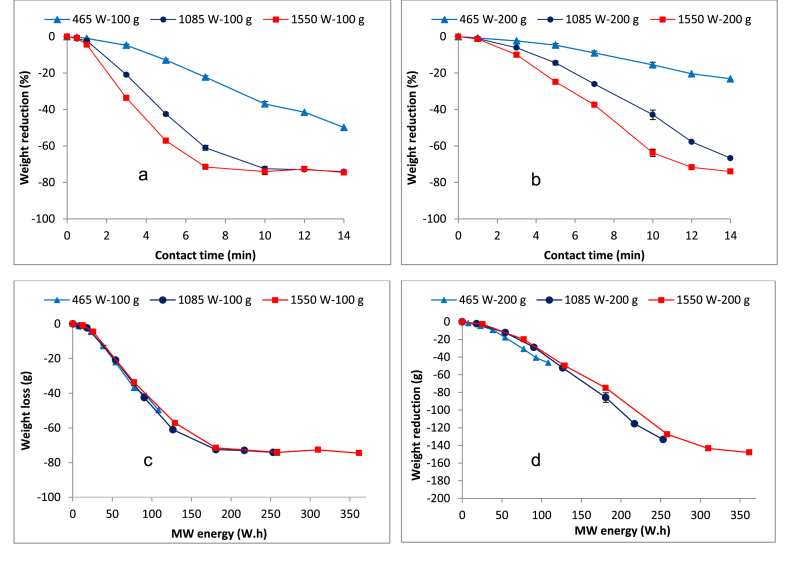
Effect of MW irradiation on sludge weight in a) 100 g sample and b) 200 g sample, and weight reduction as a function of MW energy demand on c) 100 g sample and d) 200 g sample.

Fig. 4a and b presents the profiles for weight/volume reduction in the two sample fractions evaluated. The resulting trends are strongly linked to the temperature evolution and the three phases described above (Section 3.1). The vol/wt reductions at any phase varied between both the input MW power levels and the sample sizes. The highest weight reductions at any phase in the two samples were achieved with the highest input MW power evaluated (i.e. 1550 W), while the least was at the lowest input power (i.e. 465 W). In the 100 g sample, for instance, the initial phase that lasted up to one minute achieved a weight reduction of 1.0, 2.3, and 4.5% for the 465, 1085 and 1550 W, respectively. On the other hand, respective weight reductions of 0.8, 1.1, and 1.4% for the 465, 1085 and 1550 W were attained in the 200 g sample within the one-minute contact time (i.e. initial phase). In all cases of sample fractions, substantial moisture evaporation appears to start in the subsequent second phase exhibited by a high but relatively constant weight reduction rate. The lowest vol/wt reduction for both sample fractions was observed in the final phase, which was attained in the 100 g at 1085 W and 1550 W with contact time beyond 10 min and seven minutes, respectively. However, this phase was only achieved with the 1550 W in the 200 g sample with contact time beyond 10 min.

In order to determine the energy demand during the MW treatment, the energy consumption profiles were observed (Fig. 4c and d). Like the temperature evolution and weight reduction, the energy demand profiles correspond with the three phases observed. The energy demand results (estimated by computing linear regressions on the three phases) demonstrated relatively high but varied energy demands between the two sample fractions, especially during the initial phase. For instance, the 200 g sample exhibited a higher energy demand (approximately 7 Wh per gram of weight loss or 7 kWh per kg) than the 100 g sample (approximately 6 Wh per gram or 6 kWh per kg).

### 3.4 Total and volatile solids

The VS and TS for the 100 g and 200 g sample were measured and their respective VS/TS ratio computed. From the results there was no observed change in the VS/TS ratio, which was used as an indicator to estimate the organic stability in the treated FS. For each sample fraction and the input MW power levels tested, the final VS/TS ratio was between 86 and 92%.

## 4 Discussion

### 4.1 Temperature evolution

The three distinctive temperature propagation phases observed in Fig. 1a and b are similar to those reported in the previous studies in which other types of sludge were treated using MW (Mawioo et al., 2016) and other drying methods such as convection and conduction (Bennamoun et al., 2013, Flaga, 2005). The observed initial, second, and final phases were classified as the preliminary drying phase, the essential (major) drying phase, and the final drying phase, respectively. The rapid temperature rise during the initial (preliminary) drying phase corresponds to that observed by Mawioo et al. (2016) and Yu et al. (2010) and is attributed to the high amounts of heat generated as a result of the interaction between the microwaves and the initially high concentration of the dipolar molecules (e.g. water, proteins, etc.) that are present in the wet sludge. The variability in temperature increments between the 100 g and the 200 g samples during the preliminary drying phase can be attributed to their respective initial water content. Water has a high thermal capacity and constitutes over 70% of the FS used in this study, thus the majority of the initial heat is absorbed in the water fraction of the FS. Hence, a lower temperature rise is expected in the 200 g sample, which has a higher water content (i.e. higher thermal absorption capacity) than the 100 g sample. Similar observations were reported when blackwater sludge and excess sewage sludge were heated by MW (Mawioo et al., 2016, Tang et al., 2010). The fairly constant and minimal temperature rise observed during the essential (i.e. second or major) drying phase was attributed to the possibility that the sludge may have reached the boiling point. During this phase, the unbound water is constantly evaporated from the surface of the sludge particles while being replaced by that from inside the particles (Flaga, 2005, Mawioo et al., 2016). The final (third) drying phase, as manifested in a rapid rise in the sludge temperature is a result of a more rapid evaporation of the water on the surface than it is replaced from the inside of sludge particles (Mawioo et al., 2016). Furthermore, it is evident from the results that the rate of temperature propagation is dependent both on the amount of sludge used and the input E_MW_ applied. Besides, the nature of material irradiated has influence on its temperature propagation behavior (Mawioo et al., 2016). This was demonstrated when a MW receptor material was mixed with sewage sludge to rapidly attain over 900 °C (Menéndez et al., 2002, Menéndez et al., 2005).

Generally, the results demonstrate that the MW irradiation is effective and fast in heating the FS. Previous studies demonstrated the temperature evolution rate was more rapid when material is heated with MW than the conventional heating methods (Hong et al., 2004, Menéndez et al., 2002). Therefore, if applied for the FS treatment with the temperature as the main driver for the process, the MW technology can achieve a higher throughput and smaller reactor footprint. This can greatly address the challenge of land space constraints that often affect sanitation provision in scenarios such as the urban slum and emergency settlements.

### 4.2 Pathogen reduction

#### 4.2.1 *E. coli* reduction

Generally, the results in Fig. 2 show that MW treatment can provide a rapid and effective solution for the reduction of the *E. coli* in FS. This observation has also been reported in the previous studies (Border and Rice-Spearman, 1999, Lamb and Siores, 2010, Mawioo et al., 2016), but using different media than the fresh FS, which is used in this study. The increased reduction of the *E. coli* when either the input power and/or contact time were increased is due to the resulting rise in the E_MW_, which ultimately is a key factor in the MW treatment process.

Various studies have attributed both the non-thermal (electromagnetic radiation) and the thermal (temperature) effects of the MW treatment to the destruction of microorganisms including the bacteria (Banik et al., 2003, Hong et al., 2004, Shamis et al., 2008, Tyagi and Lo, 2012, Valero et al., 2014). However, the thermal effect has been reported as the main mechanism for the destruction of bacteria with the minimum temperature for complete destruction identified at 70 °C (Hong et al., 2004, Mawioo et al., 2016, Tyagi and Lo, 2012, Valero et al., 2014). As shown in the results, generally the MW treatment achieves practically complete *E. coli* reduction within a short time duration. However, the contact time for complete bacterial reduction at given input MW power level will depend on the amount of FS treated. For instance, while *E. coli* was still detected in the 200 g FS sample when treated at 1550 W and 1085 W for one minute, it was not detected in the 100 g FS sample. Furthermore, the contact time for complete bacteria (*E. coli*) reduction for a given FS quantity can be substantially shortened when the irradiation is performed at high input MW power levels. It is also demonstrated in this study that despite the FS attaining the minimum lethal temperature for bacterial destruction, (i.e. 70 °C) *E. coli* can still be detected, for instance, when the 200 g sample was exposed 1550 W at the one minute contact time (i.e. E_MW_ = 26 Wh, T = 73 °C). This observation agrees with that from a previous study by Mawioo et al. (2016), and shows that when the temperature is rapidly escalated to the lethal level, a minimum holding time is necessary to ensure compete destruction.

#### 4.2.2 *Ascaris* egg reduction

Results from Fig. 3a1, a2, and b1, b2 demonstrate that the viability of *Ascaris lumbricoides* eggs in the FS can rapidly and effectively be reduced by the MW treatment. In this study, an energy dose of 18 Wh and 39 Wh was sufficient to produce sludge complying with the WHO guidelines of one *Ascaris* egg/g TS (i.e. approximately 2.4 LRV in this case) (WHO, 2006) in the 100 g and 200 g samples, respectively. These results agree with a previous study by Mun et al. (2009), who achieved a complete reduction of the *Ascaris lumbricoides* after a 60 s contact time at 700 W input MW power. However, they used an *Ascaris* spiked soil media with smaller sample sizes (i.e. 25 g), which is different from the fresh FS used in this study. Furthermore, an approximate 2.2 log inactivation of *Taenia taeniaeformis* eggs was achieved when a 10 g sample of cat faeces was exposed to the MW treatment for only 18 s (Conder and Williams, 1983). However, it is not clear which input MW power level was evaluated in the study. Also, it was indicated that the *Taenia taeniaeformis* eggs might be three times less resistant to the MW treatment than the *Ascaris lumbricoides* eggs (Mun et al., 2009).

Like other microorganisms, the destruction of the *Ascaris* eggs may be attributed to both non-thermal (electromagnetic) and thermal effects during the MW treatment. Similar to the *E. coli* reduction, the thermal effect appears to play the major role in the destruction as demonstrated by the significant *Ascaris* egg reduction with the temperature rise in the FS samples. According to the current study, the lethal temperature to achieve a substantial destruction of the *Ascaris* egg appears to be approximately above 70 °C. This is in agreement with a previous study that used shear viscous heating in which over 90% helminth egg reduction was reported at 70 °C (Belcher et al., 2015). The results from the two sample fractions in this study suggest that the contact time required to attain the lethal temperature for the *Ascaris* egg inactivation varies with the FS quantity and input MW power applied. For the scenarios with land space constraints, as in emergency settings, it is reasonable to choose the highest possible input MW power level so as to achieve a compact system with a small footprint. A comparison of the reduction results suggest more or less equal MW energy requirements for the destruction below the detection limit of *Ascaris lumbricoides* eggs (Fig. 3 a1 and b1) and *E. coli* (Fig. 2 a1 and b1).

The short contact times for the reduction achieved with the MW treatment demonstrated that the technology is much more rapid in *Ascaris egg* inactivation than the commonly applied conventional treatment methods, such as the sludge drying beds, composting and co-composting processes, airtight storage, and others, which take over a month to achieve complete reduction (Feachem et al., 1983, Jimenez et al., 2006, Koné et al., 2007). However, the MW technology is considered more energy intensive than those conventional alternatives.

### 4.3 Volume reduction and energy requirements

The three phases observed in the vol/wt reduction (4a and 4b) and energy profiles (4c and 4d) are described above (Section 4.1). Results from Fig. 4a and b demonstrate a low vol/wt reduction during the preliminary drying phase. This can be explained by the fact that the initial MW energy supplied is mainly used to heat the FS to the boiling point before the evaporation can start. On the other hand, a higher vol/wt reduction was achieved during the essential (major) drying phase since almost all the energy supplied at this stage is used for the evaporation of the unbound water that has low energy requirements (Mawioo et al., 2016). Furthermore, as reported in Mawioo et al. (2016), the duration of the entire essential drying phase varied with both the power input levels and the sample sizes. For instance, up to seven minutes in the 100 g sample and up to 10 min in the 200 g sample was required to conclude the essential drying when both samples were irradiated at 1550 W. Shorter durations were also realized when the input MW power was raised within the same sample size. Therefore, the quantity of the irradiated sludge and input MW power are key determinants of the duration for the essential (major) drying phase. The lowest moisture loss observed during the final drying phase is due to the fact that the free water is completely exhausted at the essential drying stage. Therefore, any moisture loss at this point is only possible by evaporating the bound water requiring more energy, which explains the low weight reduction (Mawioo et al., 2016). These trends correspond to those observed when dewatered sediment sludge and blackwater sludge were subjected to MW drying (Gan, 2000, Mawioo et al., 2016).

The current study and other previous studies (Fidjeland et al., 2013, Kengne et al., 2008, Koné et al., 2007, Mawioo et al., 2016) have shown that the water fraction constitutes over 70% of the FS weight. Consequently, evaporation of water contributes to a greater extend the FS vol/wt reduction during the drying. For example, in this study, the over 70% overall FS vol/wt reduction can strongly be linked with evaporation of water during the entire MW treatment process. As shown in the results, water is mainly evaporated during the essential drying, which can be considered the most crucial to FS vol/wt reduction. This observation corresponds with previous studies on MW treatment of various sludge types (Mawioo et al., 2016, Menéndez et al., 2002). Therefore, the optimal operation window for an FS drying MW unit can be established within the essential (major) drying phase. In the practical field applications, the level of drying can be determined based on the cost of transporting the treated sludge to the disposal facilities.

Furthermore, the results from Fig. 4c and d shows a variation in energy demand between the two sample fractions that can be linked to their differences in total water content, which needs to be heated to the boiling point before any substantial moisture loss can be realized. The 200 g sample has a higher total water content and thus higher energy demand. The essential drying phase exhibited relatively similar energy consumption rates between the two sample fractions. As reported in Mawioo et al. (2016), the energy supplied in the essential phase is mostly used in the evaporation of water. The lowest energy demand is also exhibited in this phase, which is approximately 3 Wh per gram (3 kWh per kg) of weight loss for both sample fractions. This observation agrees with Mawioo et al. (2016) and can be explained by the evaporation of the free (unbound) water, which commences once the sludge is previously heated in the preliminary phase. Based on sewage sludge, Bennamoun et al. (2013) reported energy consumptions during essential drying between 0.7–1.4 and 0.8–1.0 kWh per kg of water evaporated in the convective and conductive industrial driers, respectively. The comparatively higher specific energy consumptions attained in this study can possibly be explained by the differences in the drying units' design and the sludge types treated, and the scale of treatment equipment (Mawioo et al., 2016). A microwave reactor with adequate ventilation and insulation can probably reduce the energy demand reported in the current study. The final drying phase, which was achieved with 1085 W and 1550 W in the 100 g sample and only 1550 W in the 200 g sample, marked the highest sludge temperature and energy demand. In the case of the 100 g sample, for instance, energy demand of approximately 30 Wh was required per gram of weight loss (i.e. 30 kWh per kg). The removal of molecularly bound water at this stage requires more energy and thus the high energy demand.

Comparing the results for *E. coli* and *Ascaris* egg reduction with the vol/wt reduction, it can be observed that comparatively much higher MW energy is required to achieve substantial vol/wt reduction than that required for the complete pathogen reduction. For instance, while over 180 Wh was required to attain 70% FS vol/wt reduction, a complete *E. coli* and *Ascaris* egg reduction was achieved at 23 Wh in the 200 g sample. This suggests that when FS drying is considered in the MW reactor design, then the pathogen reduction will also take place.

### 4.4 Total and volatile solids

Based on the results for VS and TS, there was no observed reduction of the VS/TS ratio in the two MW treated sample fractions. The final VS/TS ratio ranging between 86 and 92% was higher than the 60% recommended by the European Environment Agency (Bresters et al., 1997) as the reference for the stable sludge. A previous study by Mawioo et al. (2016) reported similar results in which they observed that the temperature attained with the blackwater sludge (i.e. 127 °C) was lower than the 550 °C normally recommended for VS ignition (APHA, 1995). Similarly, a maximum temperature of approximately 134 °C was attained in the current study (Fig. 1a) which is considered very low for the VS ignition.

### 4.5 Microwave application for the treatment of faecal sludge from intensively used toilets

The results obtained in this study potentially demonstrate the technical feasibility of MW technology application for FS management in intensively used toilet facilities. The technology was found to be highly effective in the removal of both the *E. coli* and *Ascaris* eggs, which are considered most resistant in FS and are used as indicator organisms (Feachem et al., 1983, Koné et al., 2007, WHO, 2006). Relatively short contact time was required for pathogen reduction, implying that a short design retention time and small reactor footprint can be achieved. When FS drying is desired, however, expectedly longer retention times will be required depending on the initial water content in the FS and the level of drying desired. Additionally, the MW technology has relatively higher energy requirements than some conventional options (e.g. composting, anaerobic digestion, sun drying, etc.). Therefore, considering large-scale applications, the technology can be more appropriate for specific scenarios such as the urban emergencies where land space is limited and/or digging of pits is restricted leading to use of mobile toilets that require frequent emptying of fresh FS.

This study validates the MW application for the treatment of FS under the tested conditions and sets the basis for scaling up the technology and evaluating it on the basis of its performance, energy consumption, and operation. Economic analysis and comparison with other relevant technologies can also be performed. When designing a large-scale MW reactor unit for the practical field applications, a high capacity for input power (i.e. large microwave generators) should be considered to ensure rapid temperature escalations, which is important for the pathogen and sludge drying. This will ensure a smaller reactor footprint, which can easily be integrated into a compact (and if necessary) containerized system that is easy to transport and can quickly be erected and started up onsite.

## 5 Conclusions and recommendations

In this study, the evaluation of MW technology was conducted using fresh FS obtained from urine diversion toilets managed by the social enterprise Sanergy. The fresh FS was obtained from toilets spread out in two informal settlements (Mukuru and Mathare slums) in Nairobi, Kenya. The study demonstrated for the first time that MW irradiation could be applied for rapid treatment of fresh FS obtained under slum conditions. Rapid temperature escalations can be achieved in the fresh FS, which is essential for sludge sanitization and volume reduction. In addition to the *E. coli*, *Ascaris lumbricoides* eggs, which are considered among the most resistant organisms in FS, were rapidly inactivated beyond the detection limit. A volume reduction above 70% was achieved. However, more energy is required to achieve a significant volume reduction than sludge sanitization. Organic stabilization of the FS matter was expectedly not achieved with the MW treatment within the range of the test conditions in this study. The study was carried out in slum conditions, which are relatively similar to emergencies, so the results can be extrapolated to emergency settings. Further research is required to evaluate at a larger scale the performance and energy consumption of a customized MW unit under similar field conditions as tested in this study. With the large-scale unit, an economic analysis and comparison with other relevant technologies can be performed while the microbial indicators can be expanded to a wider specter of pathogens (e.g. viruses).

## Figures and Tables

**Table 1:** Characteristics of the raw FS, N = 6.

Parameter	Average	STDEV
Water content, %	74	2
Total solids, %	26	2
VS/TS ratio	0.92	0.01
TCOD (mg O2/g TS)	1.98 × 10^5^	2.8 × 10^4^
*E. coli* (CFU/g TS)	4.0 × 10^8^	8 × 10^7^
*Ascaris lumbricoides* eggs (eggs/g TS)	Not detected	–
